# Supplementary material for: Prime editing efficiency and fidelity are enhanced in the absence of mismatch repair
Source: Nat Commun. 2022 Feb 9;13:760. doi: 10.1038/s41467-022-28442-1 (PMC8828784; doi:10.1038/s41467-022-28442-1)
Supplement: Supplementary file 1 — Supplementary information [file 41467_2022_28442_MOESM1_ESM.pdf]

Supplementary Information

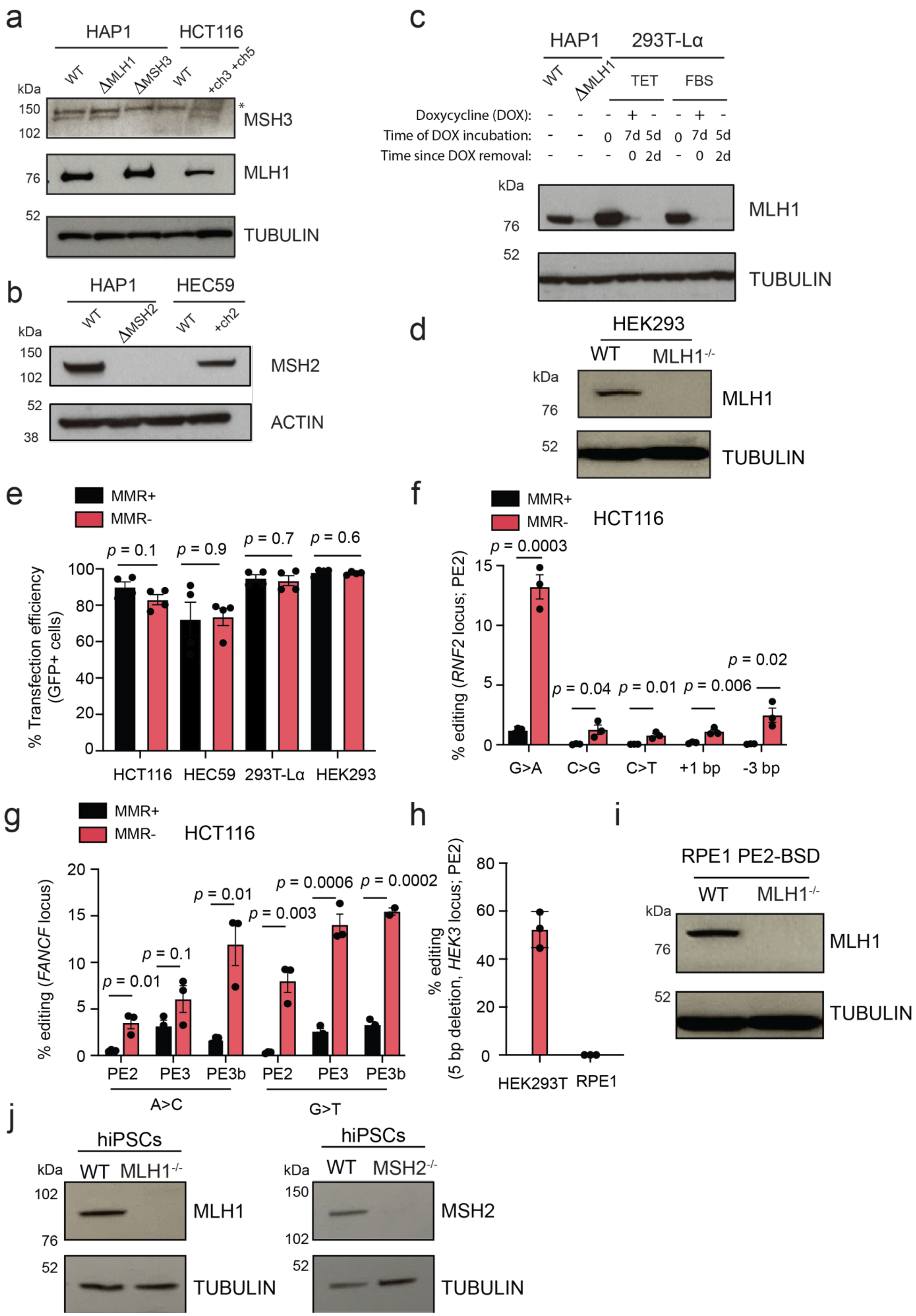

a
HAP1
HCT116
WT
ΔMLH1
ΔMSH3
WT
+ch3 +ch5
kDa
150
102
*
MSH3
76
MLH1
52
TUBULIN
b
HAP1
HEC59
WT
ΔMSH2
WT
+ch2
kDa
150
102
MSH2
52
38
ACTIN
c
HAP1
293T-Lα
WT
ΔMLH1
TET
FBS
Doxycycline (DOX): - - - + - - + -
Time of DOX incubation: - - 0 7d 5d 0 7d 5d
Time since DOX removal: - - - 0 2d - 0 2d
kDa
76
52
MLH1
TUBULIN
d
HEK293
WT
MLH1-/-
kDa
76
52
MLH1
TUBULIN
e
MMR+
MMR-
p = 0.1
p = 0.9
p = 0.7
p = 0.6
% Transfection efficiency (GFP+ cells)
HCT116
HEC59
293T-Lα
HEK293
f
MMR+
MMR-
HCT116
p = 0.0003
p = 0.04
p = 0.01
p = 0.006
p = 0.02
% editing (RNF2 locus; PE2)
G>A
C>G
C>T
+1 bp
-3 bp
g
MMR+
MMR-
HCT116
p = 0.01
p = 0.1
p = 0.01
p = 0.003
p = 0.0006
p = 0.0002
% editing (FANCF locus)
PE2
PE3
PE3b
PE2
PE3
PE3b
A>C
G>T
h
% editing (5 bp deletion, HEK3 locus; PE2)
HEK293T
RPE1
i
RPE1 PE2-BSD
WT
MLH1-/-
kDa
76
52
MLH1
TUBULIN
j
hiPSCs
kDa
WT
MLH1-/-
102
76
52
MLH1
TUBULIN
hiPSCs
kDa
WT
MSH2-/-
150
102
52
MSH2
TUBULIN

Supplementary Figure 1: Characterisation of mismatch repair-proficient cell lines. A) Immunoblot for MSH3, MLH1 and Tubulin in HAP1 (WT, ΔMLH1, ΔMSH3) and HCT116 (WT and complemented with chromosome 3 and 5) cell extracts. * denotes a non-specific band. n=1 biologically independent experiment. **B)** Immunoblot for MSH2 and Actin in HAP1 (WT, ΔMSH2) and HEC59 (WT and complemented with chromosome 2) cell extracts. n=2 biologically independent experiments. **C)** Immunoblot for MLH1 and Tubulin in HAP1 (WT, ΔMLH1) and 293T-Lα cell extracts. 293T-Lα were cultured with or without doxycycline (DOX) in 10% of Tet-approved FBS (TET) or regular FBS. n=2 biologically independent experiments. **D)** Immunoblot for MLH1 and tubulin in HEK293 WT cells, as well as an MLH1-isogenic knockout ($MLH1^{-/-}$). n=1 biologically independent experiment. **E)** Transfection efficiency of indicated cell lines, measured by flow-cytometry, after transfection of a GFP-encoding plasmid, for n=4 biologically independent experiments. **F)** Efficiency of PE2 for different mutations in the *RNF2* locus, measured in HCT116 cells complemented with chromosomes 3 and 5 (MMR+), as well as HCT116 WT (MMR-). Editing efficiency measured for n=3 biologically independent experiments. **G)** Efficiency of PE2, PE3 and PE3b after installation of an A>C or a G>T mutation in the *FANCF* locus, in HCT116 cells complemented with chromosomes 3 and 5 (MMR+), or HCT116 WT (MMR-). Editing efficiency measured for n=3 biologically independent experiments. **H)** PE2 efficiency after installation of a 5 bp deletion in the *HEK3* locus, in HEK293T cells as well as RPE1 cells. These cell lines express Cas9(H840A)-RT constitutively (PE2-BSD). Editing efficiency measured by Sanger sequencing and analysed by TIDE, for n=3 biologically independent experiments. **I)** Immunoblot for MLH1 and Tubulin in RPE1 WT cells, as well an MLH1 isogenic knockout ($MLH1^{-/-}$).These cell lines express Cas9(H480)-RT constitutively (RPE1 PE2-BSD). n=1 biologically independent experiment. **J)** Immunoblot for MLH1 (left) and MSH2 (right) in WT human induced pluripotent stem cells (hiPSCs) as well as isogenic knockouts for MLH1 ($MLH1^{-/-}$) and MSH2 ($MSH2^{-/-}$). n=2 biologically independent experiments. Statistical analysis using unpaired two-tailed Student's *t*-test across biological replicates only. Error bars reflect mean ± s.e.m. Source data are provided as a Source Data file.

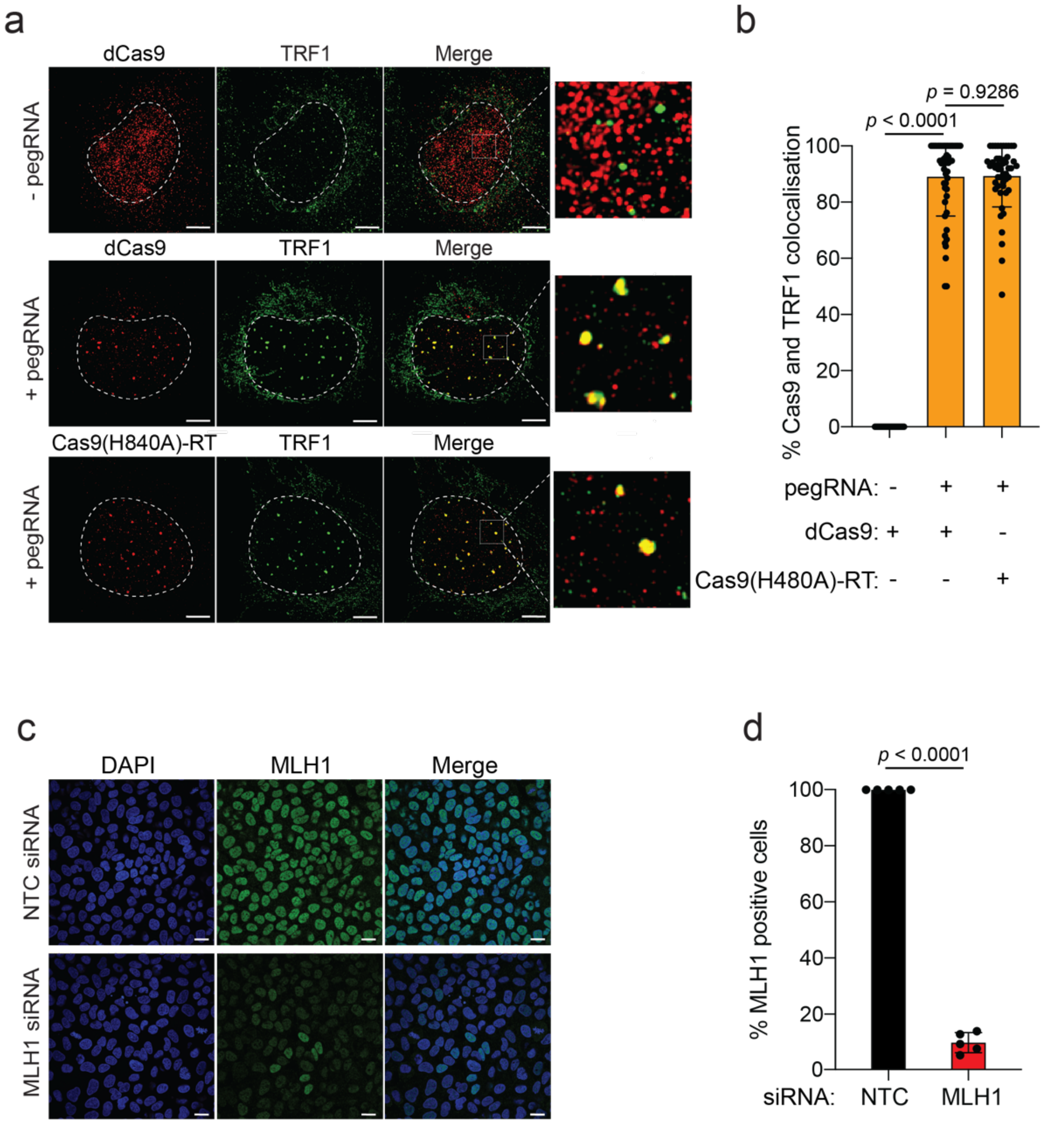

Supplementary Figure 2: Localisation of proteins to sites of active prime editing. A) Representative super-resolution images of dCas9, or Cas9(H840A)-RT, and TRF1 in U2OS cells, 24 hours following reverse transfection in the presence and absence of a pegRNA targeting telomeric repeats. n=minimum 50 cells examined over 3 biologically independent experiments. Scale bars=5 µm. **B)** Quantification of A, indicating the percentage of dCas9 and Cas9(H840A)-RT foci that co-localise with TRF1, in the presence or absence of a pegRNA targeting telomeric regions. n=minimum 50 cells examined over 3 biologically independent experiments. **C)** Representative images of MLH1 staining in U2OS cells transfected with either a non-targeting control siRNA (NTC) or an siRNA targeting MLH1. Scale bars=20 µm. n=minimum 50 cells examined over 3 biologically independent experiments. **D)** Quantification of C indicating percentage of MLH1-positive cells. n=minimum 50 cells examined over 3 biologically independent experiments. Statistical analysis using unpaired two-tailed Student's *t*-test across biological replicates only. Error bars reflect mean ± s.e.m. Source data are provided as a Source Data file.

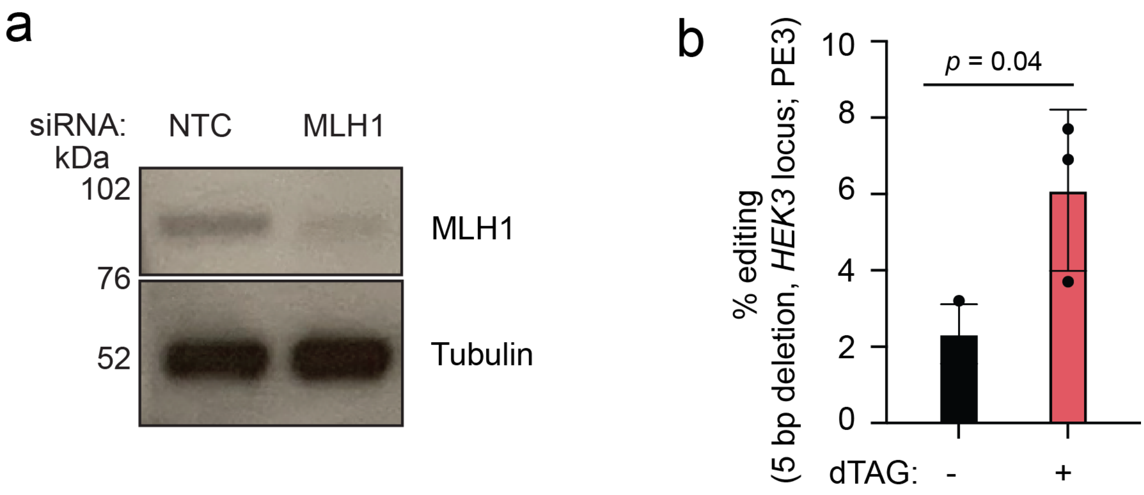

Supplementary Figure 3: Strategies to improve prime editing efficiency by transient ablation of MLH1. A) Immunoblot for MLH1 and Tubulin in HEK293 cell extracts, three days post transfection with non-targeting control (NTC) or MLH1 siRNA pools (of four siRNAs). n=3 biologically independent experiments. **B)** PE efficiency (PE3) of a 5 bp deletion in the *HEK3* locus in HAP1 dTAG-MLH1 cells in the presence and absence of 500 nM dTAG-7. Values correspond to editing efficiency, measured by Sanger sequencing and analysed by TIDE (Brinkman et al., 2014), for n=3 biologically independent experiments. Statistical analysis using unpaired two-tailed Student's *t*-test across biological replicates only. Error bars reflect mean ± s.e.m. Source data are provided as a Source Data file.

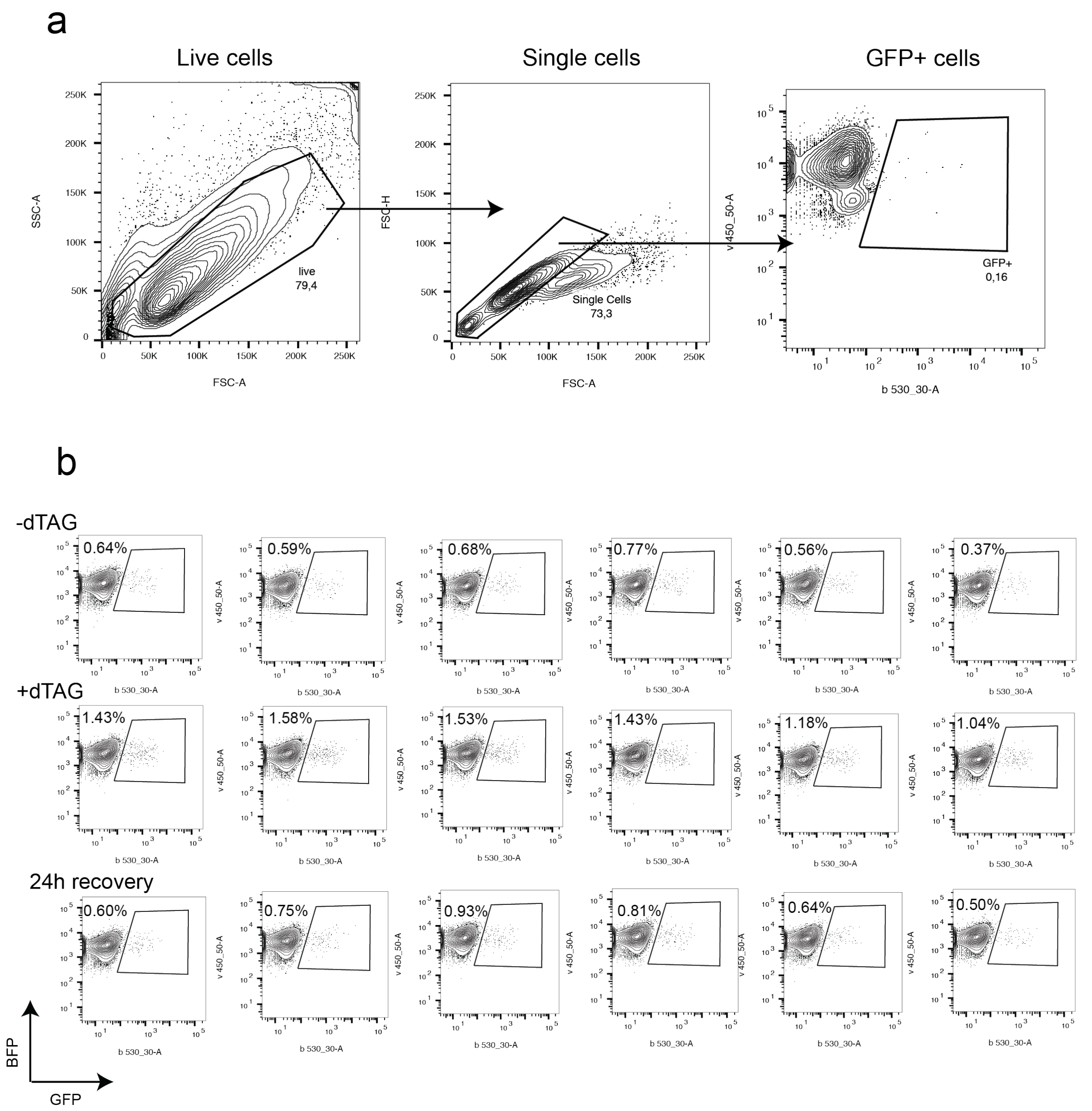

Supplementary Figure 4: Gating strategy and flow cytometry plots. A) Gating strategy to assess BFP>GFP conversion by PE2. Live cells were gated on FSC-A and SSC-A profiles. Within this gate, doublets were excluded based on the FSC-A and FSC-H profile. BFP/GFP-double positive cells were gated within single cells, using a GFP-negative sample as a control. The population within the BFP/GFP-double positive gate was used to determine the percentage of BFP>GFP conversion depicted in Figure 3C. **B)** FACS plots showing the percentage of BFP>GFP conversion by PE in dTAG-MLH1 HAP1 cells, treated with the dTAG ligand ('+dTAG'), untreated ('-dTAG'), or after 24 hours in ligand-free media ('24h recovery'). N=3 biologically independent experiments, with two technical replicates each.

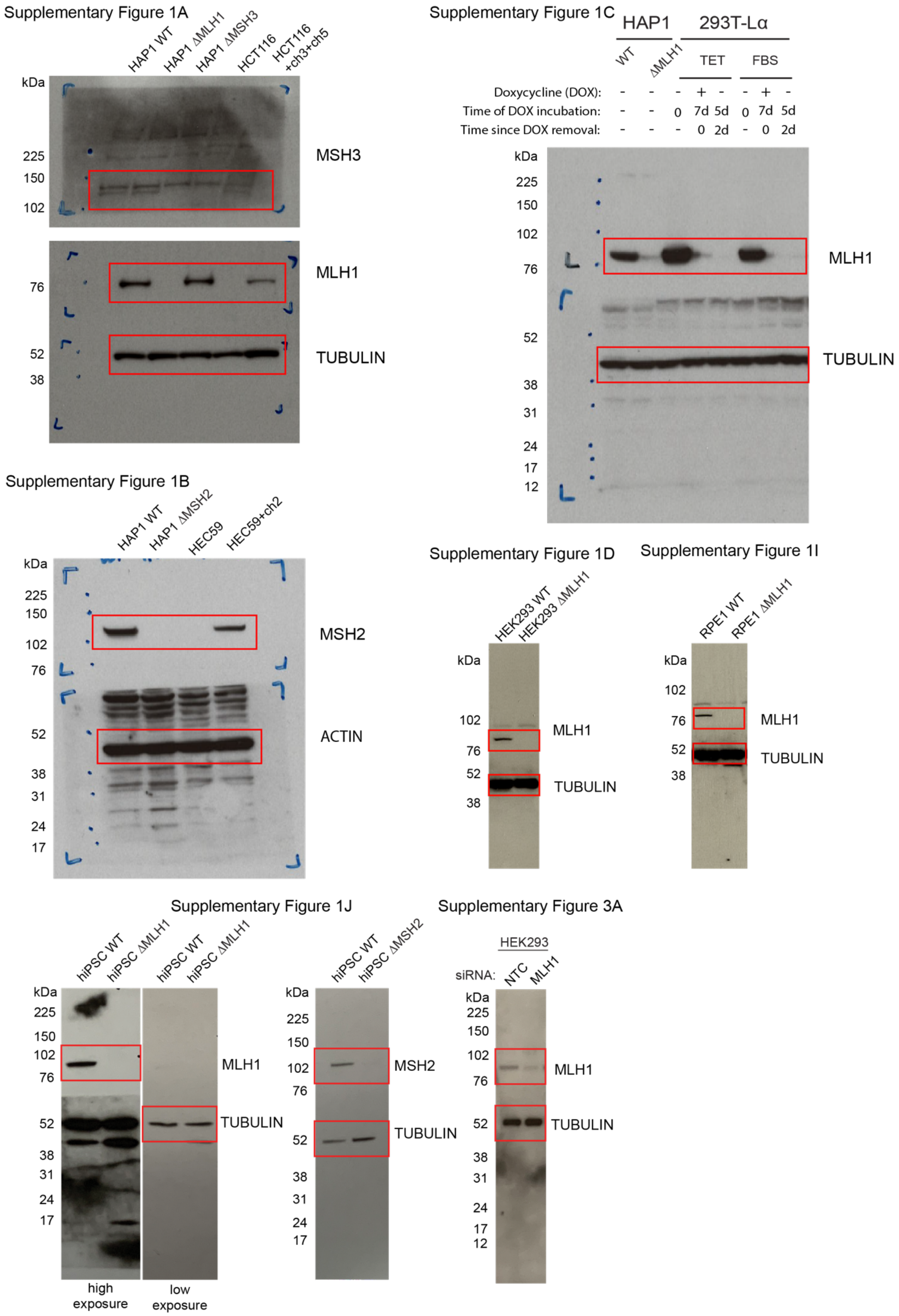

Supplementary Figure 5: Uncropped immunoblots displayed in the supplementary figures.
