# Supplementary material for: Prime editing efficiency and fidelity are enhanced in the absence of mismatch repair
Source: Nat Commun. 2022 Feb 9;13:760. doi: 10.1038/s41467-022-28442-1 (PMC8828784; doi:10.1038/s41467-022-28442-1)
Supplement: Supplementary file 2 — Reporting Summary [file 41467_2022_28442_MOESM2_ESM.pdf]

nature portfolio

Corresponding author(s): Joanna Loizou

Last updated by author(s): Jan 21st 2022

Reporting Summary

Nature Portfolio wishes to improve the reproducibility of the work that we publish. This form provides structure for consistency and transparency in reporting. For further information on Nature Portfolio policies, see our Editorial Policies and the Editorial Policy Checklist.

Statistics

For all statistical analyses, confirm that the following items are present in the figure legend, table legend, main text, or Methods section.

n/a	Confirmed	
☐	☒	The exact sample size (n) for each experimental group/condition, given as a discrete number and unit of measurement
☐	☒	A statement on whether measurements were taken from distinct samples or whether the same sample was measured repeatedly
☐	☒	The statistical test(s) used AND whether they are one- or two-sided<br>*Only common tests should be described solely by name; describe more complex techniques in the Methods section.*
☐	☒	A description of all covariates tested
☒	☐	A description of any assumptions or corrections, such as tests of normality and adjustment for multiple comparisons
☐	☒	A full description of the statistical parameters including central tendency (e.g. means) or other basic estimates (e.g. regression coefficient) AND variation (e.g. standard deviation) or associated estimates of uncertainty (e.g. confidence intervals)
☐	☒	For null hypothesis testing, the test statistic (e.g. F, t, r) with confidence intervals, effect sizes, degrees of freedom and P value noted<br>*Give P values as exact values whenever suitable.*
☒	☐	For Bayesian analysis, information on the choice of priors and Markov chain Monte Carlo settings
☒	☐	For hierarchical and complex designs, identification of the appropriate level for tests and full reporting of outcomes
☒	☐	Estimates of effect sizes (e.g. Cohen's d, Pearson's r), indicating how they were calculated

Our web collection on statistics for biologists contains articles on many of the points above.

Software and code

Policy information about availability of computer code

Data collection	Illumina MiSeq Control software v4 was used on the Illumina MiSeq sequences to collect Next Generation Sequencing data. PegRNAs were designed using the PrimeDesign software. sgRNAs were designed using the VBC score tool, except for the HAP1 screen and generation of dTAG-MLH1 cell line in which the CHOPCHOP tool was used.
Data analysis	Crispresso2 was used to analyse Next Generation Sequencing data for quantifying editing efficiency in different genomic loci. Frequency, mean and standard deviations were calculated using GraphPad Prism 8. z-stacks from immunofluorescence images were processed using Olympus 3D Deconvolution software (cellSens Dimension 3.1). FlowJo(TM) 10 was used for flow cytometry analysis. TIDE was used to analyse Sanger sequencing data.

For manuscripts utilizing custom algorithms or software that are central to the research but not yet described in published literature, software must be made available to editors and reviewers. We strongly encourage code deposition in a community repository (e.g. GitHub). See the Nature Portfolio guidelines for submitting code & software for further information.

Data

Policy information about availability of data

All manuscripts must include a data availability statement. This statement should provide the following information, where applicable:

- Accession codes, unique identifiers, or web links for publicly available datasets
- A description of any restrictions on data availability
- For clinical datasets or third party data, please ensure that the statement adheres to our policy

The sequencing data generated in this study has been deposited in the European Nucleotide Archive (EMBL-EBI; ENA) under accession code PRJEB47501 (https://www.ebi.ac.uk/ena/browser/view/PRJEB47501). Source data are provided with this paper.

Field-specific reporting

Please select the one below that is the best fit for your research. If you are not sure, read the appropriate sections before making your selection.

☒ Life sciences ☐ Behavioural & social sciences ☐ Ecological, evolutionary & environmental sciences

For a reference copy of the document with all sections, see nature.com/documents/nr-reporting-summary-flat.pdf

Life sciences study design

All studies must disclose on these points even when the disclosure is negative.

Sample size	Sample sizes were determined based on literature precedence for genome editing (eg. Anzalone et al., Nature 2019). For immunofluorescence experiments aimed to acquire at least 50 images per condition.
Data exclusions	No data was excluded.
Replication	All experiments were performed at least in two independent but mostly with three biological replicates, with technical replicates. The exact number of replicates for each experiment is indicated in the figure legends.
Randomization	Mammalian cells used in this study were grown under identical conditions. No randomization was used.
Blinding	For manual microscopy, data were collected randomly by choosing at least 3 regions. The investigators who analyzed data were blind to group allocation. For other experiments, the investigators who collected and analysed the data were not blinded to group allocation.

Reporting for specific materials, systems and methods

We require information from authors about some types of materials, experimental systems and methods used in many studies. Here, indicate whether each material, system or method listed is relevant to your study. If you are not sure if a list item applies to your research, read the appropriate section before selecting a response.

Materials & experimental systems

n/a	Involved in the study	
☐	☒	Antibodies
☐	☒	Eukaryotic cell lines
☒	☐	Palaeontology and archaeology
☒	☐	Animals and other organisms
☒	☐	Human research participants
☒	☐	Clinical data
☒	☐	Dual use research of concern

Methods

n/a	Involved in the study	
☒	☐	ChIP-seq
☐	☒	Flow cytometry
☒	☐	MRI-based neuroimaging

Antibodies

Antibodies used	Immunoblotting: MLH1 (554073, BD Pharmigen, 1:1000), MSH2 (ab52266, Abcam, 1:1000), MSH3 (ab69619, Abcam, 1:1000), Tubulin (3873, Cell Signaling, 1:5000), ß-Actin (A5060, Sigma, 1:5000), HRP-conjugated goat anti-mouse (115-035-003, JacksonImmuno, 1:2000) and HRP-conjugated goat anti-rabbit (111-035-003, JacksonImmuno, 1:2000).<br><br>Immunofluorescence: anti-Cas9 (Cell Signalling, 14697), anti-TRF1 (Abcam, ab1423), anti-MLH1 (ThermoFisher, A300-015A), anti-GFP (Abcam, ab6556), goat anti-mouse Alexa Fluor® 568 (A-11004, ThermoFisher, 1:2000) and goat anti-rabbit Alexa Fluor® 488 (A-11034, ThermoFisher, 1:2000).
Validation	For immunoblotting, the antibodies were validated using knock-out cell lines for the respective proteins.<br>The antibody anti-Cas9 (Cell Signalling, 14697) has been validated for immunofluorescence by the manufacturer in 293 cells.<br>The antibody TRF1 (Abcam, ab1423) has been validated for immunocytochemistry by the manufacturer and for immunofluorescence in previous publications [Hu, MH. et al Nucleic acids Research (PMID: 27923993)], [Douarre, C. et al Nucleic acids Research (PMID: 23396447)] and [Fakhoury, J. et al Journal of Cell Science (PMID: 20427319)].<br>The anti-MLH1 (ThermoFisher, A300-015A) has been validated for immunofluorescence by the manufacturer in U-251 MG cells and by us here using U2OS cells treated with a pool of siRNAs against MLH1.<br>The anti-GFP antibody (Abcam, ab6556) has used in over 1000 publications and been validated for immunofluorescence by the manufacturer in primary hippocampal neurons and rat glial cells and by us using transfected U2OS in the presence and absence of a GFP encoding plasmid.<br>The HRP-conjugated goat anti-mouse (115-035-003, JacksonImmuno) has been validated for immunoblotting by the manufacturer and was used in over 1400 publications.

The HRP-conjugated goat anti-rabbit (111-035-003, JacksonImmuno) has been validated for immunoblotting by the manufacturer and was used in over 1500 publications.
The goat anti-mouse Alexa Fluor® 568 (A-11004, ThermoFisher) has been validated for immunofluorescence by the manufacturer in HeLa cells and was used in 290 publications.
The goat anti-rabbit Alexa Fluor® 488 (A-11034, ThermoFisher) has been validated for immunofluorescence by the manufacturer in HeLa cells and was used in over 850 publications.
Further validation can be found in the manuscript.

Eukaryotic cell lines

Policy information about cell lines

Cell line source(s)

HAP1 (Horizon Discovery)

U2OS, RPE1, HCT116 and HEK293 (ATCC)

HEC59 and HEC59+chromosome 2 are a non-commercial isogenic pair generated by Minoru Koi at the University of Michigan and first described in this paper:
Umar A, Koi M, Risinger JI, Glaab WE, Tindall KR, Kolodner RD, Boland CR, Barrett JC, Kunkel TACorrection of hypermutability, N-methyl-N'-nitro-N-nitrosoguanidine resistance, and defective DNA mismatch repair by introducing chromosome 2 into human tumor cells with mutations in MSH2 and MSH6. (1997) Cancer Res 57: 3949-3955. Cancer research. 57. 3949-55.

HCT116+chromosome3+5 and 293T-Lalpha were provided by Prof.Josef Jiricny (ETH Zurich, Switzerland)

hiPSCs were a gift from Prof Nik-Zainal (University of Cambridge, UK)

Authentication

All cell lines have been authenticated based on morphological criteria. Additionally, U2OS, RPE1 and HEK293 have been authenticated by STR profiling.

Mycoplasma contamination

All cell lines were tested negative for mycoplasma.

Commonly misidentified lines
(See ICLAC register)

No commonly misidentified cell lines were used.

Flow Cytometry

Plots

Confirm that:

- [x] The axis labels state the marker and fluorochrome used (e.g. CD4-FITC).
- [x] The axis scales are clearly visible. Include numbers along axes only for bottom left plot of group (a 'group' is an analysis of identical markers).
- [x] All plots are contour plots with outliers or pseudocolor plots.
- [x] A numerical value for number of cells or percentage (with statistics) is provided.

Methodology

Sample preparation

25,000 dTAG-MLH1 HAP1 cells were seeded in 48-well plates (Corning) with and without 500 nM of dTAG-7 (R&D Systems). 24h post-seeding, cells were transfected with 0.5uL of Lipofectamine 2000 and 200 ng of pCMV-PE2 and 50 ng of the pegRNA targeting the BFP locus, converting it to GFP. Some conditions were kept without dTAG-7 ('-'), with dTAG-7 ('+') and some conditions previously treated with d-TAG7 where kept without it ('24h recovery'). BFP to GFP conversion was measured 96 hours after transfection by flow cytometry analysis.

Instrument

Measurements were performed iusing the BD LSRFortessa(TM) X-20 Cell Analyser. FACS sorting was performed using a BD FACSMelody(TM) Cell Sorter

Software

BD FACSDiva(TM) software was used for acquisition. Analysis was performed using FlowJo(TM) 10

Cell population abundance

10,000 cells within the 'single cell' gate were acquired.

Gating strategy

Live cells were gated based on their FSC-A against SSC-A profile. Single cells were gated within live cells based on their FSC-A against FSC-H profile. Within this, GFP+ cells were analysed.

- [x] Tick this box to confirm that a figure exemplifying the gating strategy is provided in the Supplementary Information.
