# Supplementary material for: Prime editing efficiency and fidelity are enhanced in the absence of mismatch repair
Source: Nat Commun. 2022 Feb 9;13:760. doi: 10.1038/s41467-022-28442-1 (PMC8828784; doi:10.1038/s41467-022-28442-1)
Supplement: Supplementary file 4 — Description of Additional Supplementary Files [file 41467_2022_28442_MOESM4_ESM.pdf]

**Title:** Supplementary Data 1
**Description:** Frameshift mutations in collection of 32 knockout HAP1 cell lines, covering all DNA repair pathways.

**Title:** Supplementary Data 2:
**Description:** Sequences of pegRNAs, sgRNAs and primers used throughout the study.
