# Supplementary material for: Prime editing efficiency and fidelity are enhanced in the absence of mismatch repair
Source: Nat Commun. 2022 Feb 9;13:760. doi: 10.1038/s41467-022-28442-1 (PMC8828784; doi:10.1038/s41467-022-28442-1)
Supplement: Supplementary file 5 — Supplementary data 1 [file 41467_2022_28442_MOESM5_ESM.docx]

| **IGene** | **Sequence (5’ to 3’)** | | **Indel (bp)** |
| --- | --- | --- | --- |
| *WT* | ATTCAGATCCAAGACAATGGCACCGGGATCAGGGTAAGTA | | -2 |
| *ΔMLH1* | ATTCAGATCCAAGACAATGGCACCGGG--CAGGGTAAGTA | |  |
| *WT* | TTTTCGACCGGGGCGACTTCTATACGGCGCACGGCGAGGA | | -2 |
| *ΔMSH2* | TTTTCGACCGGGG--ACTTCTATACGGCGCACGGCGAGGA | |  |
| *WT* | GATCAAGCCTATTCTCGTATTTGATGGATGTACTTTACCTTC | | -8 |
| *ΔEXO1* | GATCAAGCCTATT--------TGATGGATGTACTTTACCTTC | |  |
| *WT* | AAACGGTCCAAAAGCATCTATACGCCGCTAGAATTACAATA | | 1 |
| *ΔMSH3* | AAACGGTCCAAAAAGCATCTATACGCCGCTAGAATTACAAT | |  |
| *WT* | AGGCATCCGTGTAAGTTGCACCAATCAGCTTGGACAAGGA | | 1 |
| *ΔPMS2* | AGGCATCCGTGTAAGTTGCACCAATCAAGCTTGGACAAGG | |  |
| *WT* | TTGGTTTGGGCCAAGATGGAGGGTTACCCCTGGTGGCCTT | | -14 |
| *ΔMSH6* | TTGGTTTGGGCCAAGA--------------TGGTGGCCTT | |  |
| *WT* | AGGACAGCCGGCACCAGCGAATCAGACTTCCACATTTGGA | | -11 |
| *ΔAPTX* | AGGACAGCCGG-----------CAGACTTCCACATTTGGA | |  |
| *WT* | TGCCACGCCATGCCCACCTGCGCTTTTACACGGCCCCGCCT | | 1 |
| *ΔNEIL1* | TGCCACGCCATGCCCACCTGCGCTTTTTACACGGCCCCGCC | |  |
| *WT* | AGTATGCCAAGTCCAACAGAAGTACGTGCAAGGGGTGTAT | | 1 |
| *ΔPARP1* | AGTATGCCAAGTCCAACAGAAGTACGTTGCAAGGGGTGTA | |  |
| *WT* | AGGCCGCCATGAGCAAACGGAAGGCGCCGCAGGAGACTC | | -5 |
| *ΔPOLB* | AGGCCGCCATGAGCAAACGGAAG-----GCAGGAGACTC | |  |
| *WT* | CTGTTGTCCAGGCTAAGTTGACAACCACTGGCCAGGTGAC | | +185 |
| *ΔLIG3* | CTGTTGTCCAGGCGGGCGCCTGGGCTTCCAACAAGGCTTT  TCTTCTCCAACACGTCCCGCCTCCCCCCACACACAAAGTAA  AAGAAAAACAAACCAAAAACGACTTTCAAAGAGACAGGTG  TCGGGTTCAAGGTCATACGGGAAGGTACCAGGACCTTGGT  TGGGAACATGACAGGAGACTACCACTCAAGCCTCGCTTAA  GTTGACAACCACTGGCCAGGTGAC | |  |
| *WT* | CAGTCATCCTGGGGGTTCAGCAGATGGTCCAGCAAAAAAG | | -10 |
| *ΔXPC* | CAGTCATC------------CAGATGGTCCAGCAAAAAAG | |  |
| *WT* | TGTAAAAGCAGCCCCAAAGATAATTGACACAGGAGGAGGC | | -4 |
| *ΔXPA* | TGTAAAAGCAGCCCCAAAGATAA----CACAGGAGGAGGC | |  |
| *WT* | GGTTTTTGTCCGCACGCCAAACGGGTTTGGAGGACCCTCTT | | -14 |
| *ΔERCC8* | GGTTTTTGTCCGCACGCCAAACGGG--------------TT | |  |
| *WT* | GGCCAGCCCTGCTGCACATCGACCGACATCAGATCCAGGC | | -5 |
| *ΔERCC6* | GGCCAGCCCTGCT-----TCGACCGACATCAGATCCAGGC | |  |
|  | |  |  |
| *WT* | TTCTCTCAGCCGGGATGGTTGCCTCTAGCGTGGGACAGAT | | 2 |
| *ΔFANCA* | TTCTCTCAGCCGGGATGGTTGCCTCTATAGCGTGGGACAG | |  |
| *WT* | CATTATTGCCAGATTTCTTGCAGACTCCGAAGGAAGTTGTA | | -5 |
| *ΔBLM* | CATTATTGCCAGATTTCTTGCAGA-----AAGGAAGTTGTA | |  |
| *WT* | GGGCCCAGAATACATAAGTAGCCGCATGGCTGGCGGAGGC | | 2 |
| *ΔRAD52* | GGGCCCAGAATACATAAGTAGCCGCATATGGCTGGCGGAG | |  |
| *WT* | CAGGGAGCCCCACCCATGTACCAGCTCTATAAGCGGACGT | | -17 |
| *ΔPRKDC* | CAGGG-----------------AGCTCTATAAGCGGACGT | |  |
| *WT* | GTTATTACACTTACTGATGGTCATTCAGCATGGACTGGGAC | | -2 |
| *ΔXRCC4* | GTTATTACACTTACTGATGGTCATTCA--ATGGACTGGGAC | |  |
| *WT* | AGTTTAACCATACAGCAAGTAAACGACCTTTTAGACTCAAT | | 1 |
| *ΔLIG4* | AGTTTAACCATACAAGCAAGTAAACGACCTTTTAGACTCAA | |  |
| *WT* | ATAATCTCCTTCGCCCATTGTTGAAGGACGCTGCTCACCCT | | -4 |
| *ΔXLF* | ATAATCTCCTTCG----TTGTTGAAGGACGCTGCTCACCCT | |  |
| *WT* | ATCAGGCTCAGATTCGTTCTCGGGAAGCGGCGGTGACAGC | | 1 |
| *ΔPOLQ* | ATCAGGCTCAGATTCGTTCTCGGGAAGTCGGCGGTGACAG | |  |
| *WT* | GCCTGTGGAGAGGTAAAGCTGGCTTTCGAGAGGAAAACAT | | -10 |
| *ΔCHK2* | GCCTGTGGAGAGGTAAA----------GAGAGGAAAACAT | |  |
| *WT* | CCACTTCCTCAGTTTCGGCACTCAAGCCGCCTGGAACGGAG | | -1 |
| *ΔPOLE* | CCACTTCCTCAGTTTCGGCACTCAAGC-GCCTGGAACGGAG | |  |
| *WT* | TGAGCAGGGTCTGCACGAAATAAAGCTCCTGGGCAAGGGG | | -8 |
| *ΔMGMT* | TGAGCAGGGTCTGCACGAAATAAAGC--------AAGGGG | |  |
| *WT* | AGGCCCTCAGCCCGAATGTGCCGCCAGCTAGGGCCTGCTG | | -4 |
| *ΔALKBH2* | AGGCCCTCAGCCCGAATGTGCCGCC----AGGGCCTGCTG | |  |
| *WT* | AATTTTTAGTGGAGTTGCCATCTATGTTAATGGATACACAG | | -28 |
| *ΔREV1* | AATTTTTAGTGG----------------------------G | |  |
| *WT* | AAGTCAACCAACGAATTGAAAATATGATGCAACAAAAAGC | | -4 |
| *ΔPOLK* | AAGTCAACCAA----TTGAAAATATGATGCAACAAAAAGC | |  |
| *WT* | GAGAAGACCTGACCCGCTACAGAGAAATGTCTTATAAGGT | | -1 |
| *ΔPOLI* | GAGAAGACCTGA-CCGCTACAGAGAAATGTCTTATAAGGT | |  |
| *WT* | CTTCTACTGGCACAAGTTCGTGAGTCCCGTGGGAAAGCTA | | -1 |
| *ΔPOLH* | CTTCTACTGGCACAAGTTCGTGAGTCC-GTGGGAAAGCTA | |  |

sgRNA sequence

Protospacer Adjacent Motif (PAM)
